# Measuring tactile sensitivity and mixed-reality-assisted exercise for carpal tunnel syndrome by ultrasound mid-air haptics

**DOI:** 10.3389/fnins.2024.1319965

**Published:** 2024-01-18

**Authors:** Mehmet Akif Akdağ, Ahmet Kıvanç Menekşeoğlu, Hatice Seğmen, Berk Gözek, Merve Damla Korkmaz, Burak Güçlü

**Affiliations:** ^1^Institute of Biomedical Engineering, Boğaziçi University, İstanbul, Türkiye; ^2^Department of Physical Medicine and Rehabilitation, University of Health Sciences, İstanbul Kanuni Sultan Süleyman Training and Research Hospital, İstanbul, Türkiye; ^3^Department of Neurology, University of Health Sciences, Istanbul Kanuni Sultan Süleyman Training and Research Hospital, İstanbul, Türkiye

**Keywords:** touch, psychophysics, somatosensory system, carpal tunnel syndrome, mid-air haptics, neuropathy, nerve conduction studies, tactile feedback

## Abstract

**Introduction:**

Carpal tunnel syndrome (CTS) is the most common nerve entrapment neuropathy, which causes numbness and pain in the thumb, the index and middle fingers and the radial side of the ring finger. Regular hand exercises may improve the symptoms and prevent carpal tunnel surgery. This study applied a novel ultrasonic stimulation method to test tactile sensitivity in CTS and also a mixed-reality-assisted (MR-assisted) exercise program which measured hand movements and provided haptic feedback for rehabilitation.

**Methods:**

Twenty patients with mild unilateral CTS took part in the experiments. A mid-air haptics device (Ultrahaptics STRATOS Explore) was used to apply amplitude-modulated ultrasound waves (carrier frequency: 40 kHz) onto the skin to create tactile stimulation mechanically. Participants performed a two-alternative forced-choice task for measuring tactile thresholds at 250-Hz modulation frequency. They were tested at the index fingers and the thenar eminences of both hands. Additionally, 15 CTS patients used an MR-assisted program to do hand exercises with haptic feedback. Exercise performance was assessed by calculating errors between target and actual hand configurations. System Usability Scale (SUS) was adopted to verify the practical usability of the program.

**Results:**

Thresholds at the thenar eminences of the affected and healthy hands were not significantly different. While the thresholds at the healthy index fingers could be measured, those of the affected fingers were all higher than the stimulation level produced by the maximum output from the ultrasound device. In the exercise program, a significant positive correlation (ρ = 0.89, *p* < 0.001) was found between the performance scores and the SUS scores, which were above the criterion value established in the literature.

**Discussion:**

The results show that thenar tactile sensitivity is not affected in mild CTS as expected from the palmar cutaneous branch of the median nerve (PCBm), but index finger threshold is likely to be higher. Overall, this study suggests that mid-air haptics, with certain improvements, may be used as a preliminary test in the clinical setting. Moreover, the device is promising to develop gamified rehabilitation programs and for the treatment follow-up of CTS.

## Introduction

Ultrasound mid-air haptics is a novel technology that takes advantage of contactless tactile sensations by vibrating the air and superficial skin layers. Most other haptic devices require the user to wear equipment, which may disrupt the sensations especially during movement because of skin-contact decoupling. Another novel technology, electrovibration displays, for example, only work efficiently if the user scans his/her finger(s) across the tactile surface with more or less constant force (Jamalzadeh et al., 2020). Therefore, ultrasound mid-air haptics and complementary hand tracking devices may improve user engagement during interaction with digital contents (Limerick et al., 2019). Because of improvements in human-computer interaction, they have attracted significant recent attention in the literature (e.g., Iwamoto et al., 2008), for application areas such as automotive user interfaces, virtual reality (VR), augmented reality (AR), mixed reality (MR) applications, and games (Georgiou et al., 2018; Harrington et al., 2018; Martinez et al., 2018). Martinez et al. (2018) developed several mid-air haptic algorithms for rendering 3D shapes. By changing the movement pattern of an ultrasonic focus point, various different sensations can be evoked. Hwang et al. (2017) designed AirPiano, which is a VR piano playing experience that provides mid-air haptic feedback. HaptiGlow technique uses visual light cues to help users position hands over mid-haptic devices to optimize tactile sensations (Freeman et al., 2019). Similar techniques with auditory cues would also be helpful in navigation panels, especially for those with visual disabilities. Although mid-air haptics technology has found many application areas as described in the literature, clinical use has not been tested as yet. Our main aim was to apply this technology to measure tactile sensitivity in patients with carpal tunnel syndrome (CTS) and explore possible rehabilitative use by an MR-assisted exercise program.

Ultrasound is the term for sound waves with frequency higher than what humans can hear, which is in the range of 20 Hz to 20 kHz, but varies between individuals and with normal aging. In mid-air haptics technology, the sense of touch is created by ultrasound waves that impinge on the skin. Due to the mechanical impedance mismatch, a single portable transducer is unable to create detectable vibrations in the skin; therefore, phased array focusing with many transducers is used to generate the required stimulation. Based on the transducer source location, each output is phase-shifted to achieve summation at the target location. A focal ‘point’ at the target is actually similar to an ellipsoid with the current technology, and its exact shape can vary (elongation, distortion) based on device limitations and the target location (Rakkolainen et al., 2020). The size of the focal point is related to the wavelength, and thus to the carrier wave frequency in the constant medium of air (speed of sound: 346 m/s at 25°C). Higher frequency yields a smaller focal point, which is ~9 mm for the device used in this study at 40 kHz (Ultrahaptics STRATOS Explore; Kirby, 2023). Carrier wave frequency largely depends on the transducer material, its geometry, and how it is excited. Fixed- and variable-frequency transducers exist commercially for various applications, such as medical devices for diagnostics, surgical devices, industrial automation as emitter/detector pairs and robotic sensing.

Since 40-kHz mechanical vibrations created in the skin cannot be detected psychophysically by humans (Güçlü, 2021), the carrier wave needs to be modulated. The application programming interface (API) for the Ultrahaptics device supports amplitude modulation (AM) and spatio-temporal modulation (STM). In AM, discrete focal points can be generated with adjustable intensity at desired target locations; STM generates a single focal point which can move back and forth along a curve at the target. The Ultrahaptics device includes the Leap Motion Controller, which uses two infrared cameras for optical hand tracking (Ultraleap, 2020). Its advanced algorithms create a 3D ball-and-stick model of the hand and ensures a continuous and flowing experience for mid-air haptics. For example, as the hand moves over the Ultrahaptics device, the ultrasonic excitation is rendered in such a way that one can feel the simulation of a 3D object surface with points and dents.

CTS is the most common peripheral nerve entrapment syndrome, which occurs due to the compression of the median nerve within the carpal tunnel (Atroshi et al., 1999; Rempel and Diao, 2004). Via the carpal tunnel, the palmar sides of the thumb, index, middle fingers, and of radial half of the ring finger receive sensory innervation from the median nerve, as well as the corresponding nail beds and distal halves of the dorsal sides. Motor innervation is supplied to the first and the second lumbrical muscles and the muscles of the thenar eminence. CTS patients may experience tingling throughout the day as their symptoms increase, particularly while engaging in specific activities like chatting on the phone, reading a book or newspaper, or driving (Zyluk and Walaszek, 2012; NIH, 2022). If untreated, the muscles that are located around the base of the thumb may atrophy over time (Genova et al., 2020). Contributing factors to CTS are repetitive motion, injury or trauma, arthritis, pregnancy, hormonal changes, obesity, and genetics (Dyer et al., 2008; Osterman et al., 2012; Burton et al., 2014; Padua et al., 2016).

In addition to simple clinical tests for the diagnosis of CTS [e.g., Tinel test (Padua et al., 2016), Phalen test (Sawaya and Sakr, 2009)], electrophysiological testing is routinely performed as a nerve conduction study (NCS; Graham, 2008; Rosario and De Jesus, 2020). Ultrasound imaging (Yoshii et al., 2020) and magnetic resonance imaging (MRI) have good sensitivity and specificity, and are especially useful for cases with clinical symptoms but no impaired electrophysiological results (Jarvik et al., 2004; Bland, 2005; Wilder-Smith et al., 2006). Patients with mild CTS can do regular hand exercises, which may improve their symptoms and avoid carpal tunnel surgery (Akalin et al., 2002; Muller et al., 2004; Horng et al., 2011). On the other hand, those who are diagnosed with CTS are often unmotivated to exercise regularly, because they find it tedious and tiresome. Novel technologies may be helpful in that respect.

In this study, we utilized the mid-air haptics technology both for the measurement of tactile sensitivity and for creating an MR environment to motivate CTS patients for hand exercise. This novel technology provided a practical setup for a simple psychophysical experiment, albeit with some incomplete results due to the limitations of the device system software. The main hypothesis was reduced sensitivity at the index finger of the affected hand, but no difference of sensitivity at the thenar eminence. The palmar cutaneous branch of the median nerve (PCBm) provides sensory innervation to the thenar eminence, and the central and radial aspects of the palm, but this branch does not travel through the carpal tunnel. As such, the detection thresholds, which could be measured in CTS patients, were reliable and suggest that mid-air haptics may be used as a preliminary test in the clinical setting. Furthermore, the MR-assisted program was a motivating and objective platform for hand exercise with an acceptable System Usability Scale (SUS) score.

## Materials and methods

### Participants

Nineteen female and one male participants (age range: 33–61) took part in the psychophysical experiments. Another group of 15 female participants (age range: 33–66) performed the MR assisted hand exercise. Based on the overall clinical exam, all participants were diagnosed with unilateral mild CTS; seven had CTS in their left hand, and the rest in the right hand. The experiments were approved by the Clinical Research Ethics Committee of the Health Sciences University Kanuni Sultan Süleyman Training and Research Hospital (application number: 2021.06.196). All participants signed consent forms, and they did not have any other neurological or dermatological conditions.

### Electrodiagnosis

The participants were initially tested by electroneuromyography (ENMG) performed by a specialist (H. Seğmen) using a standard clinical device (Neuropack S1 MEB-9400; Nihon Kohden, Japan). They lay in a comfortable posture at room temperature and with skin surface temperature of >32°C. Compound muscle action potentials (CMAP) and sensory nerve action potentials (SNAP) of the median and ulnar nerves were assessed. The median nerve was stimulated ~8 cm proximal to the recording site, which was over the abductor pollicis brevis muscle to measure the median CMAP. Orthodromic stimulation was applied at the middle finger to measure the median SNAP at the wrist. Onset latency, baseline-to-peak amplitude, and conduction velocity were calculated for each measurement. The data were analyzed according to standard reference values (Bland, 2000; Kimura, 2013; Sasaki et al., 2022). Grade 0 indicates no electrodiagnostic abnormality. In Grade 1 (very mild) CTS, orthodromic sensory conduction velocity (SCV) is >40 m/s and distal motor latency (DML) is <4.5 ms. Other more sensitive tests are used to verify Grade 1 CTS [e.g., radial/ulnar comparison, segmental studies, double peak in ring finger test (Uncini et al., 1989; Padua et al., 1997; Bland, 2000)]. In Grade 2 (mild) CTS, orthodromic SCV is <40 m/s and DML is <4.5 ms; therefore, a sensory slowing is observed. In Grade 3 (moderate) CTS, SNAP is preserved with motor slowing and DML is between 4.5 and 6.5 ms. In Grade 4 (severe) CTS, SNAP is absent, but CMAP is preserved; DML is between 4.5 and 6.5 ms. In Grade 5 (very severe) CTS, SNAP is absent and DML is >6.5 ms. In Grade 6 (extremely severe) CTS, both SNAP and CMAP are absent appreciably.

### Experimental setup

**Figure 1:**
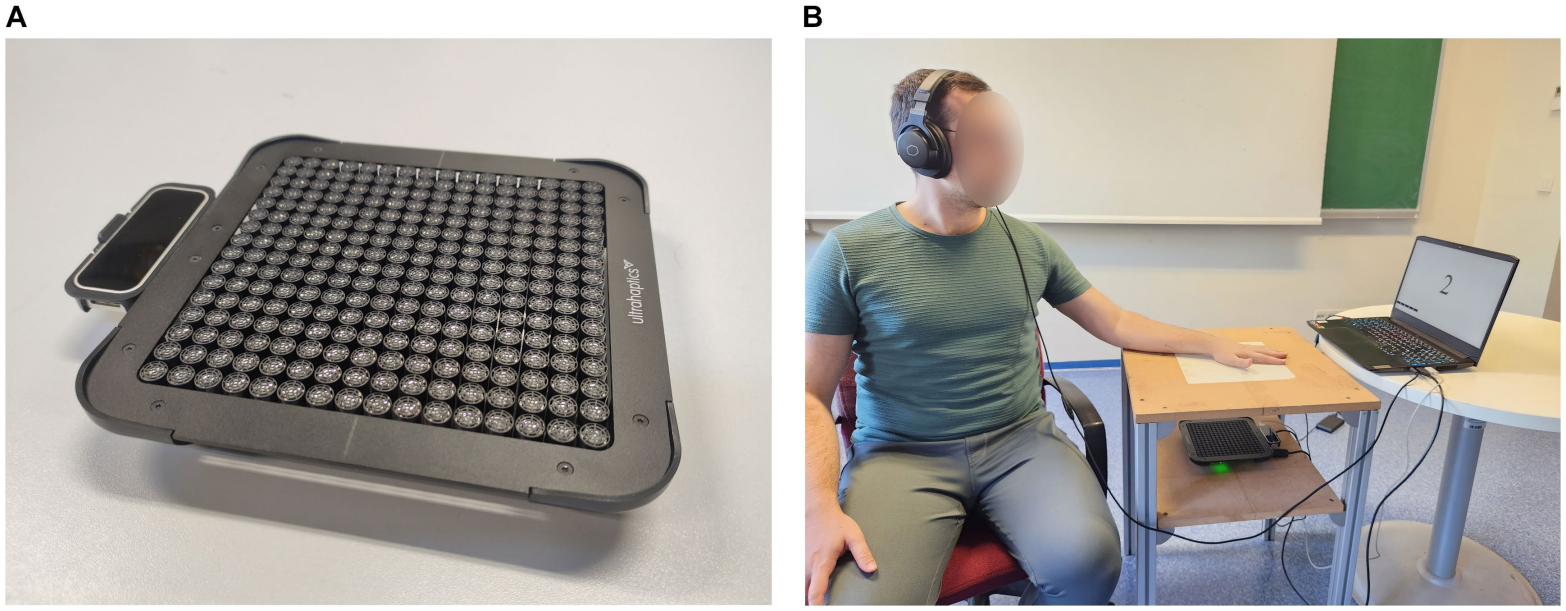
Mid-air haptics device and the experimental setup. **(A)** The mid-air haptics device (Ultrahaptics STRATOS Explore) consists of an array of ultrasound emitters. Also attached to the system is a hand tracking device (Leap Motion Controller) with infrared light emitters and cameras. **(B)** The device was placed on a stand with an adjustable distance to hand platform. The hand platform included a Plexiglas cover with different diameter holes for exposing the stimulus location on the skin to amplitude modulated ultrasonic waves. The device was controlled by specialized software on a personal computer. During the psychophysical experiments, the participants sat in a comfortable chair and wore headphones with white noise to mask auditory cues from the device. The task intervals were indicated on the computer screen. The participant responses were acquired verbally and entered to the computer by the experimenter.

The ultrasound actuated mid-air tactile stimuli were generated by the Ultrahaptics STRATOS Explore device (Ultraleap Ltd., United Kingdom; Figure 1A). For programming, its software development kit (SDK) Version 2.6.5 was used. The device also has the Leap Motion Controller attached for hand tracking. For activating the hand tracking and the ball-and-stick hand models (see below) Leap Motion SDK Version 4 was used. The suggested optimal distance for stimulation is ~20 cm (Raza et al., 2019); therefore, we placed it on a stand with an adjustable height to find the exact position (Figure 1B). At the upper platform of the stand, a Plexiglass cover is used to allow for different sized openings. Since the shape of the focal point is not exactly a sphere, the cover prevents stimulation by any side lobes or stray energy due to hardware and software imperfections. The cover for the thenar has an opening with a circular diameter of 55 mm; the cover for the digit stimulation has a similar opening with a diameter of 23 mm. Any sound cues generated by the device during ultrasonic transduction were masked by white noise presented to the participant’s ears through headphones.

### Tactile stimuli and psychophysical procedure

**Figure 2:**
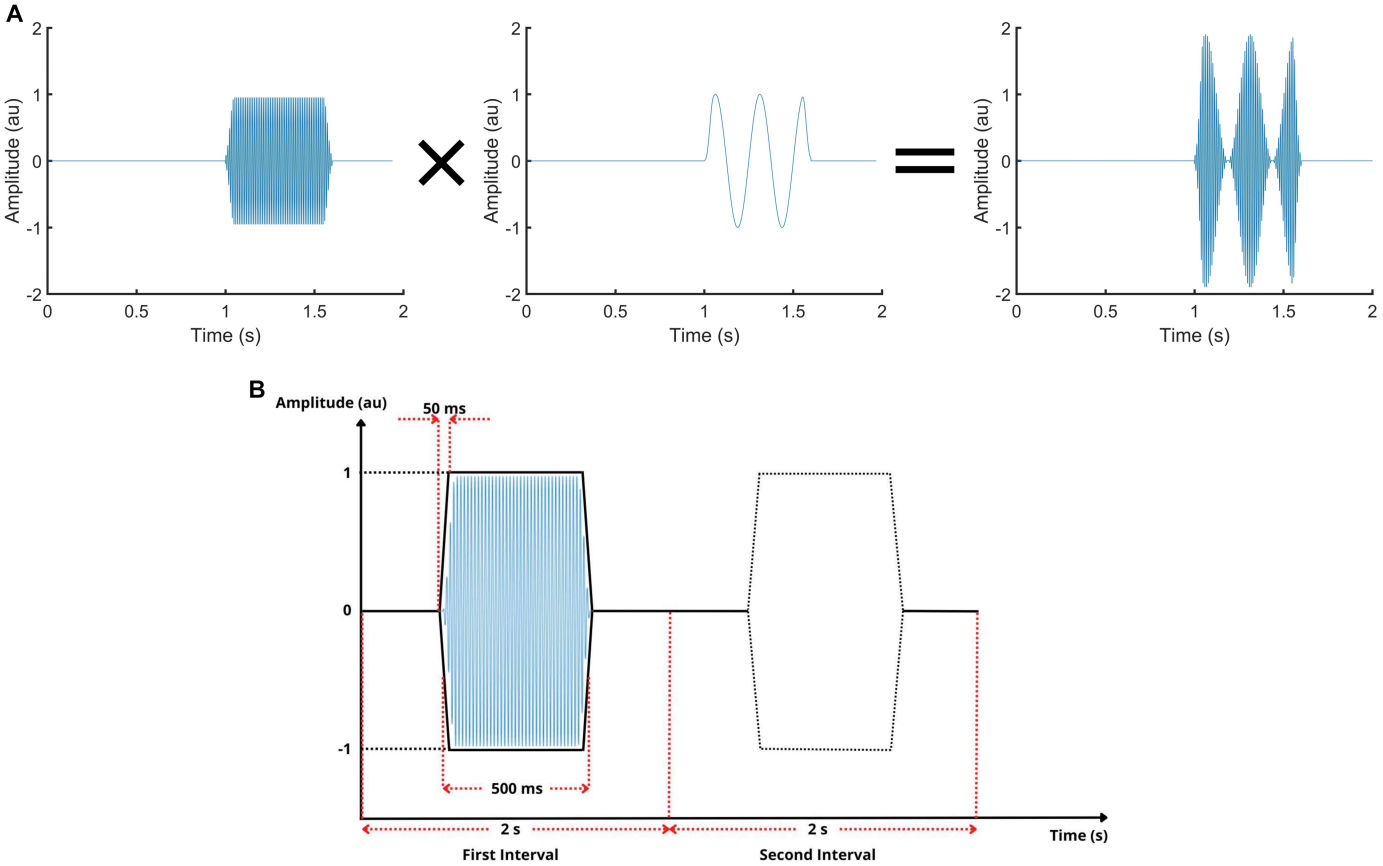
Stimulus waveform and the psychophysical task. **(A)** The ultrasonic carrier wave (left panel) is modulated by the vibrotactile waveform (middle panel) to produce an amplitude-modulated ultrasonic signal (right panel). This signal is emitted from the ultrasonic transducers in the mid-air haptics device, and produces a mechanical stimulation on the skin, similar to vibrotactile stimulation by a mechanical shaker (see text). In the figure, the frequencies of the signals are given much lower for drawing clarity. **(B)** In the two-alternative forced-choice task, the vibrotactile stimulus is presented randomly in one of the 2-s time intervals. The participant selects the interval in which he/she feels the stimulus. The vibrotactile stimulus (caused by the amplitude-modulated ultrasonic wave) is a burst of sinusoidal vibration with 250-Hz frequency and 0.5-s duration at half-power points. It is windowed by cosine-squared envelopes with 50-ms rise/fall times. The amplitude of the stimulus is represented in arbitrary units (au) set by the device.

The ultrasonic carrier wave at 40 kHz was amplitude modulated by bursts of sine waves as shown in Figure 2A. Although we did not measure mechanical displacements on the skin surface, previous calibration studies on deformable surfaces have shown that the AM ultrasonic acoustic radiation pressure causes mechanical vibrations on such surfaces (Chilles et al., 2019). The frequency component with the highest magnitude is at the frequency of the modulating signal. Therefore, within the scope of the current study, we consider and refer to the modulating waves as vibrotactile stimuli (e.g., Güçlü and Öztek, 2007) and constructed them as such for the ultrasonic mid-air haptics device. The duration of the stimulus was 0.5 s as measured between its half-power points; and it had 50-ms cosine-squared rise and fall envelope (Figure 2B). The windowing prevents additional harmonics in the vibrotactile stimulus which was at the fundamental frequency of 250 Hz. Humans are most sensitive to vibrotactile frequencies within 200–300 Hz (Güçlü, 2021). The amplitude of the stimulus was adjusted in arbitrary units (au) according to the API of the ultrasonic device.

The psychophysical procedure consisted of a two-alternative forced-choice task, which allows a criterion-free detection threshold measurement. The vibrotactile stimulus was randomly presented in one of the two time intervals (duration: 2 s, Figure 2B) at each trial. The participant verbally selected the interval in which he/she felt the stimulus; the intervals were visually cued on the computer screen. The amplitude of the stimulus changed at each trial according to participant’s responses in an adaptive tracking method, with an up-down rule that yielded threshold level at 75% correct probability of detection (Zwislocki and Relkin, 2001). This up-down rule increases the stimulus level one step for every incorrect response, and decreases it one step for every three correct responses which are not necessarily consecutive. Initially the step size was 0.1 au; and it became 0.02 au after the first reversal. The tracking was stopped if the stimulus level stayed within ±0.02 au for 20 trials. The detection threshold was recorded as the middle stimulus level in that final sequence of trials. The participant did not receive feedback regarding the correctness of his/her responses.

The procedure was actualized by a custom-made C# code developed for the Ultrahaptics API. The API requires a computer sound file (.wav format) to be played by the transducers during every trial. These files were prepared offline in MATLAB (Ver. R2022a; The MathWorks, Inc., United States) for every possible condition and recalled from the hard disk by the C# code during an experiment. Absolute thresholds were measured at the distal pads of both index fingers and at the thenar eminences of the participants in a session of about 1 h, including instructions and several training trials, but without repetitions. The 250-Hz vibrotactile stimulus is expected to activate the Pacinian psychophysical channel (Güçlü, 2021).

### MR-assisted hand exercise

**Figure 3:**
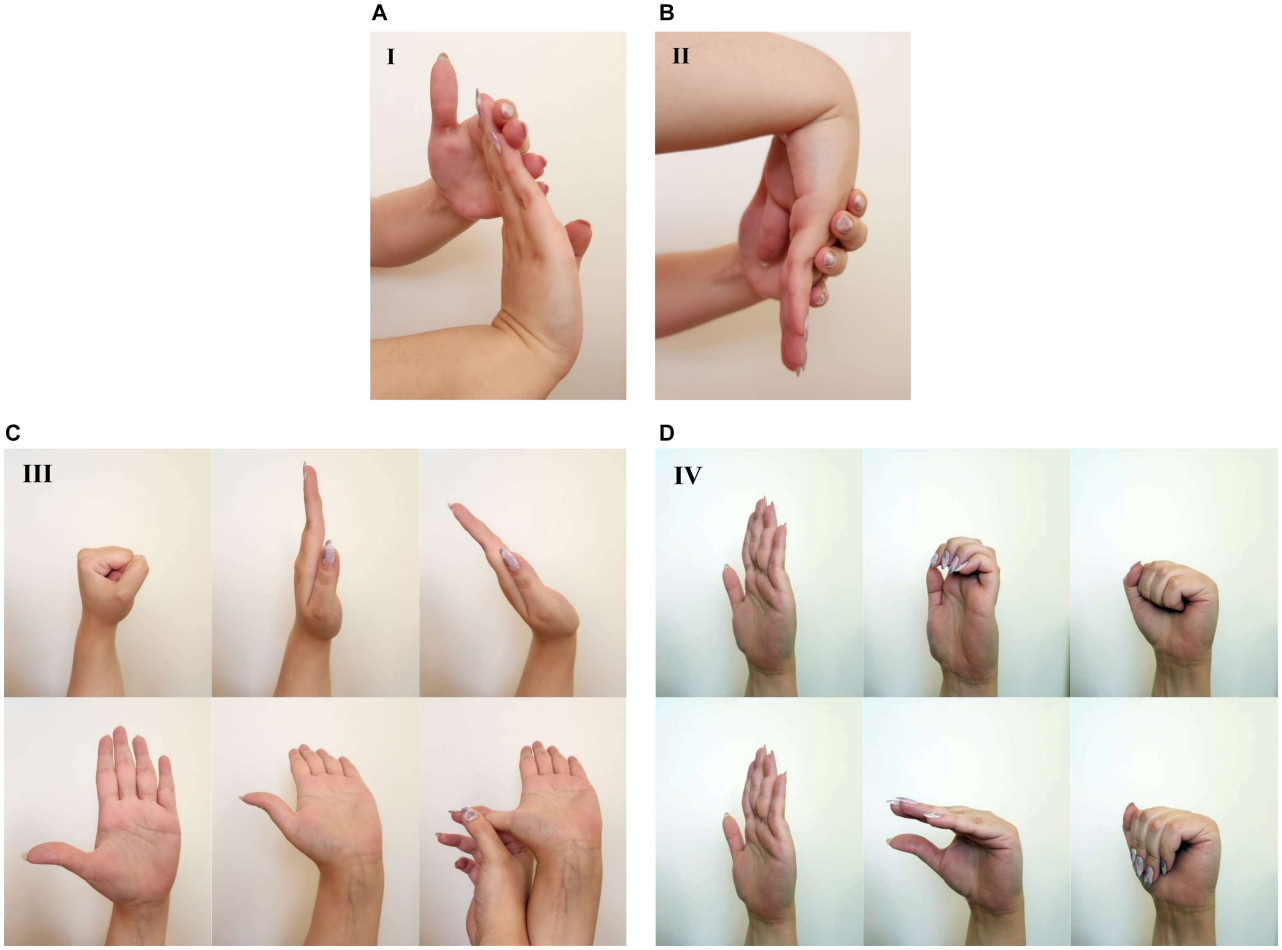
Hand exercises used in the MR-assisted program. **(A)** Wrist Extension Stretch (1 pose). **(B)** Wrist Flexion Stretch (1 pose). **(C)** Medial Nerve Glides (6 poses applied sequentially from left to right at each row from top to bottom). **(D)** Tendon Glides (6 poses applied sequentially from left to right at each row from top to bottom). See text and Table 1 for the scoring of the hand poses.

The hand exercise program was developed in the Unity game engine Ver. 2020.3.27 (Unity Technologies, United States). Within this program, the participants completed a series of hand exercises consisting of four sections (Figure 3) while holding their hand(s) directly above the mid-air haptics device and the hand-tracking cameras without using the Plexiglas covers mentioned above. The program was developed according to the guidelines of the American Academy of Orthopedic Surgeons (AAOS, 2022) and the hand exercises were similar to those used in the conservative treatment of CTS (Akalin et al., 2002; Muller et al., 2004; Horng et al., 2011). In each section, there are hand poses which need to be maintained for a certain minimum amount of time to complete the exercise. After completion of each pose, or if the participant fails the criterion, he/she is informed by visual feedback on computer screen and via the mid-air haptics feedback and the program moves either to the next pose, to the next repetition or to the new section. The performance of the participant was assessed according to how long the pose was maintained in seconds (t). The scoring scheme of the program is given in Table 1. Failure of the criterion resulted in zero score for that pose. For the current study, the score did not increase for holding the pose more than the criterion time; therefore, t was either 0 or equal to the criterion time. However, we kept t as a variable for future modifications and applications. For ease of comparison, the performance scores were later normalized with the total maximum score (330) to the range of 0–100.

**Table 1:** Scoring for the hand poses in the MR-assisted hand exercise program.

Exercise section	Poses	Criterion	Repetitions	Score formula	Max. score
I. Wrist Extension Stretch	1	15 s	5	1 × t × 5 = 75	75
II. Wrist Flexion Stretch	1	15 s	5	1 × t × 5 = 75	75
III. Medial Nerve Glides	6	5 s	3	6 × t × 3 = 90	90
IV. Tendon Glides	6	3 s	5	6 × t × 5 = 90	90

Each pose must meet the time criterion to be successful. In the current study, the pose duration (t) in the score formula was constant and equal to the criterion.

At the beginning of each pose, the mid-air haptics device was actuated for a suprathreshold 2-s tactile feedback at 250 Hz such that the hands started within the ideal zone of tracking by the Leap Motion Controller (~20 cm above the transducer surface). Although the hand tracking is quite robust to hand location changes, the participants were instructed to keep their hands mainly around the initial zone at all times. This allowed a reliable haptic feedback during the exercises as well. After each exercise pose (successful or unsuccessful), the participant was presented a 0.5-s burst of vibrotactile stimulus at 250 Hz via the mid-air haptic device. Then, he/she prepared the hands for the next pose such that the pose cycle started again.

**Figure 4:**
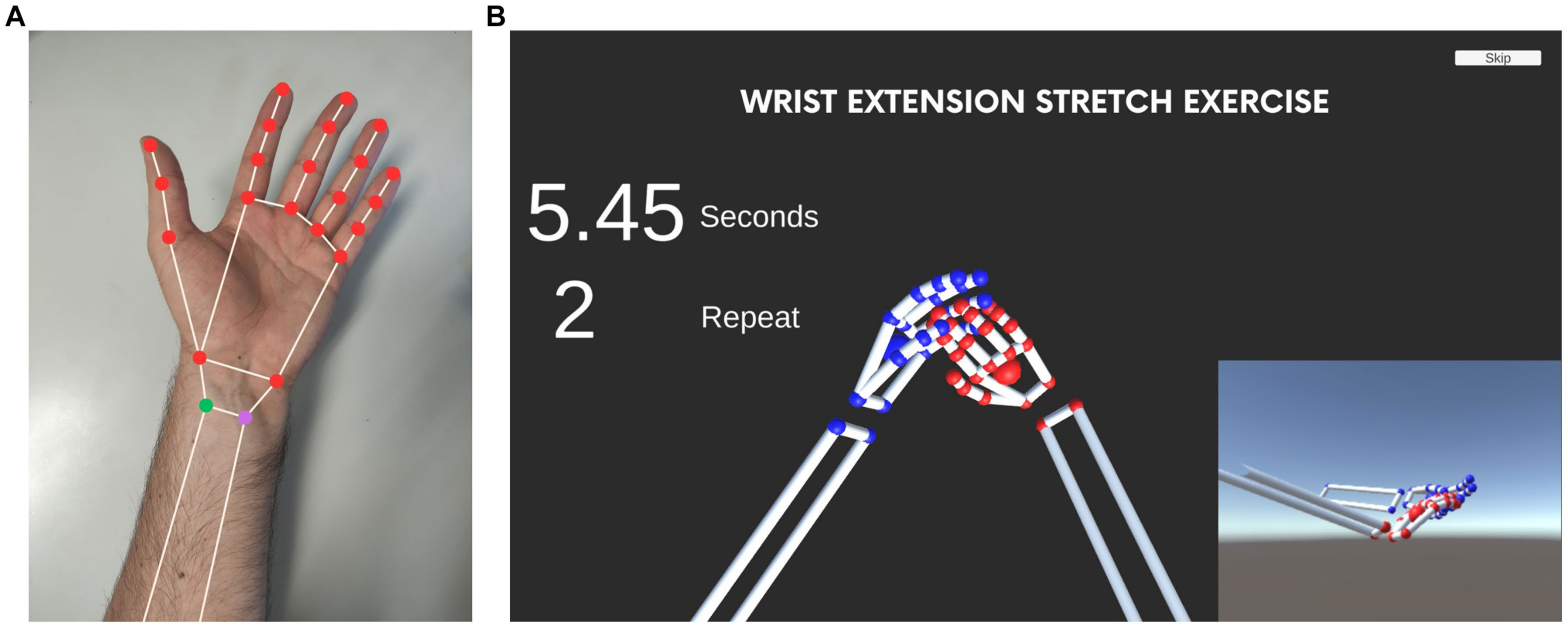
MR-assisted hand exercise program with mid-air haptic feedback. **(A)** Ball-and-stick model generated by the hand tracking part of the mid-air haptics device. The model is continuously updated as the hand moves during the exercise. **(B)** Computer screen of the hand exercise. The participants are able to see the ball-and-stick model as they perform the hand poses. A counter shows the amount of time left to satisfy each pose criterion. The repetition number is also indicated. Tactile/haptic feedback is given at the beginning and the end of each pose.


The hand tracking software of Leap Motion Controller creates real-time 3D ball-and-stick models for the hands (Figure 4A), and the model coordinates are accessed in Unity. Before the MR-assisted exercise, the software was calibrated for each pose by every participant individually, which is highly important because of the large anatomical differences between individuals (e.g., hand size, phalangeal lengths). As such, every participant generated the target poses for the registration of the coordinates of the balls given in Figure 4A. The tracking software normalizes each hand model according to its virtual reference frame, and the coordinates of red balls (*n* = 21) move with respect to the green one which is considered as fixed. In the exercise program, the distances of the red balls to the green one are calculated in real time to determine the error to the target pose. The root-mean-square error (RMSE) is defined as:

(1) $$\mathrm{RMSE}=\sqrt{\frac{1}{n}\overset{n}{\underset{i=1}{\sum }}{\left(d_{Ti}-d_{\mathrm{Ai}}\right)}^{2}}$$

Equation (1) calculates the differences between the distance of every *i*th red ball to the green ball in the target model (*d_Ti_*) and the actual model (*d_Ai_*) during the pose. Preliminary tests showed that *RMSE* < 5 cm for the normalized ball-and-stick model in the Unity virtual environment is adequate to consider the pose as maintained. The program kept the time that the pose was maintained by using a visual counter on the computer screen. The participant could see his/her hands and the screen simultaneously for an MR experience. An example computer screen during the program is shown in Figure 4B.

### System Usability Scale

In addition to the performance scores calculated as in Table 1 for the MR-assisted hand exercise program, the participants completed the questionnaire for the System Usability Scale (SUS). SUS scores in the range of 0–100 provide an industry-accepted metric for assessing the usability of websites and various products, especially new computer systems (Brooke, 1996). In the questionnaire, participants answer 10 questions with each having five response choices that represent how strongly the participant agrees or disagrees. Although SUS is very simple, it yields useful results quickly. Odd-numbered questions inquire about the system’s advantages, even-numbered questions inquire about its drawbacks. The SUS score of 68 is approximately the median (50th percentile) for many previously tested systems; therefore, new systems with SUS above 68 may be considered to be above average.

### Statistical analyses

The measurements were reported as means and standard deviations (mean ± std). Statistical comparisons between the psychophysical absolute thresholds, and for exercise and SUS scores were performed in MATLAB by using Student’s t-tests. The association between the performance in the hand exercise program and the SUS score was tested among participants by Pearson correlation.

## Results

### Electrodiagnostic measurements

In the CTS patient group who participated in the psychophysical study (*n* = 20), there were robust SNAP and CMAP preserved. Mean and standard deviations of the baseline-to-peak amplitudes were 12.2 ± 5.8 mV (SNAP) and 7.6 ± 1.7 mV (CMAP). Half of the patients was of Grade 1 with SCV as 41.5 ± 1.1 m/s and DML as 3.5 ± 0.3 ms. The other half was of Grade 2 with SCV as 36.0 ± 2.9 m/s and DMS as 3.6 ± 0.2 ms. In brief, the patients had very mild and mild grades of CTS according to only electrodiagnostic measurements. The overall average data are given in Table 2.

**Table 2:** Electrodiagnostic results of the participants with CTS.

Recording	Parameter	Mean ± STD	Unit
Median SNAP (distal)	Baseline-to-peak amplitude	12.2 ± 5.8	mV
	Onset latency	2.9 ± 0.4	ms
	Conduction velocity (SCV)	38.7 ± 3.6	m/s
Median CMAP (distal)	Baseline-to-peak amplitude	7.6 ± 1.7	mV
	Onset latency (DML)	3.6 ± 0.3	ms
	Conduction velocity	56.9 ± 5.0	m/s

SNAP, sensory nerve action potential; CMAP, compound muscle action potential; SCV, sensory conduction velocity; DML, distal motor latency.

### Psychophysical thresholds

**Figure 5:**
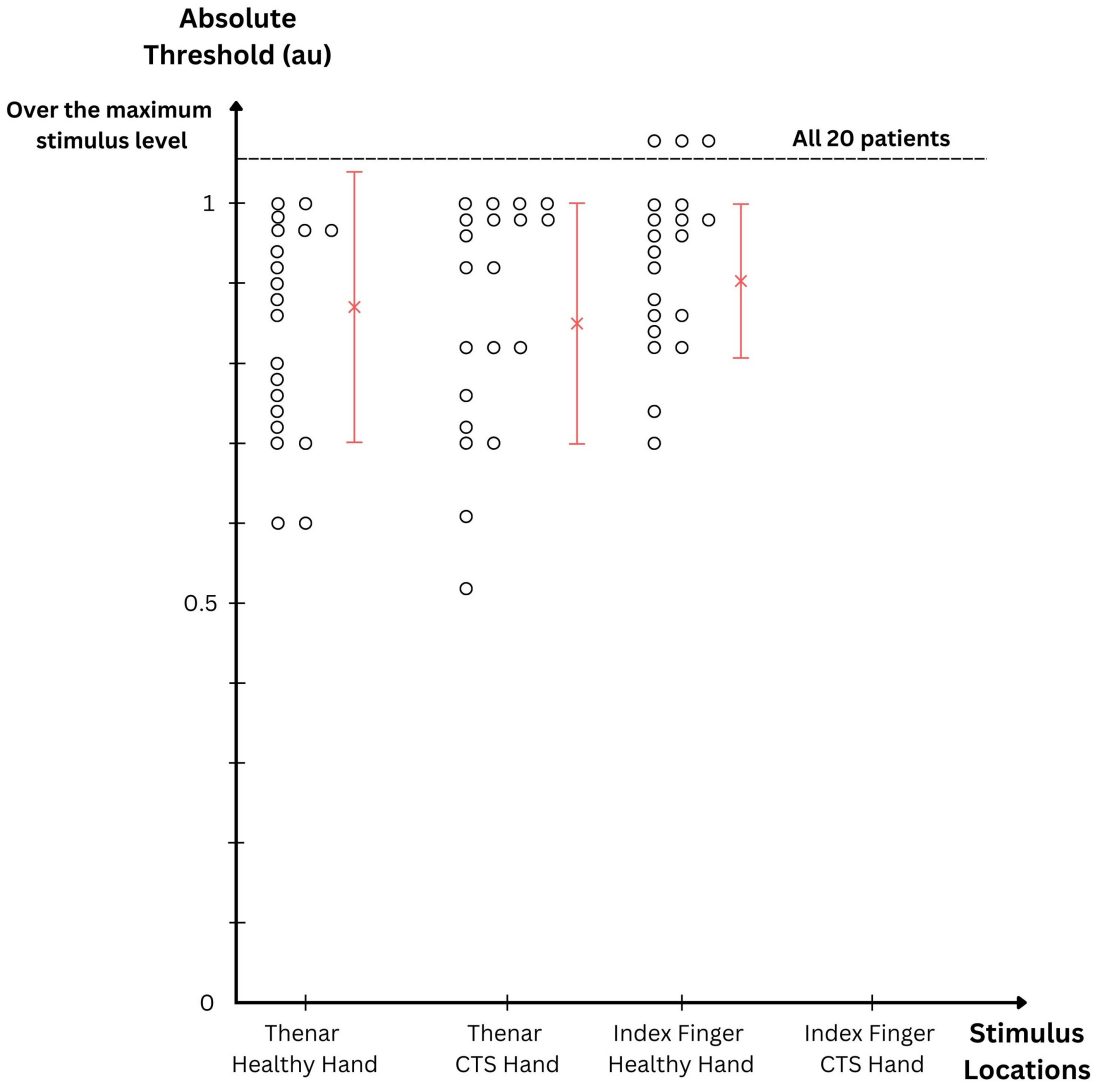
Psychophysical results of the participants with CTS. Absolute detection thresholds of 250-Hz vibrotactile stimulus are plotted for different stimulus locations. The vibrotactile stimulus originated from amplitude-modulated ultrasonic waves impinging on skin surface.

Psychophysical absolute thresholds (at 250-Hz modulation frequency) were measured with the ultrasonic mid-air haptics device at four skin locations: thenar eminences of healthy and CTS-affected hands, distal pads of index fingers of healthy and CTS-affected hands. Figure 5 shows absolute thresholds based on the arbitrary units of the device output. Data are shown for individual participants, as well as the means and standard deviations.

Thenar eminence receives sensory innervation mainly from the PCBm. Since this branch does not pass through the carpal tunnel, it was expected that both hands had similar absolute thresholds. As such, they were 0.87 ± 0.16 au and 0.85 ± 0.15 au for the healthy and affected hands, respectively. There was no statistical difference between the thenar thresholds from both hands [paired *t*(19) = −0.669; *p* = 0.511].

Absolute thresholds from the index fingers were somewhat higher. The adaptive tracking procedure did not converge with three participants while measuring the threshold from the healthy hand. The mean and standard deviation from the rest of the participants is 0.90 ± 0.09 au for the index finger of the healthy hand. On the other hand, for the CTS-affected hand, no thresholds could be measured within the output range of the device. This implies that they were above 1 au, the maximum level allowed in the device API.

### MR-assisted hand exercise

**Figure 6:**
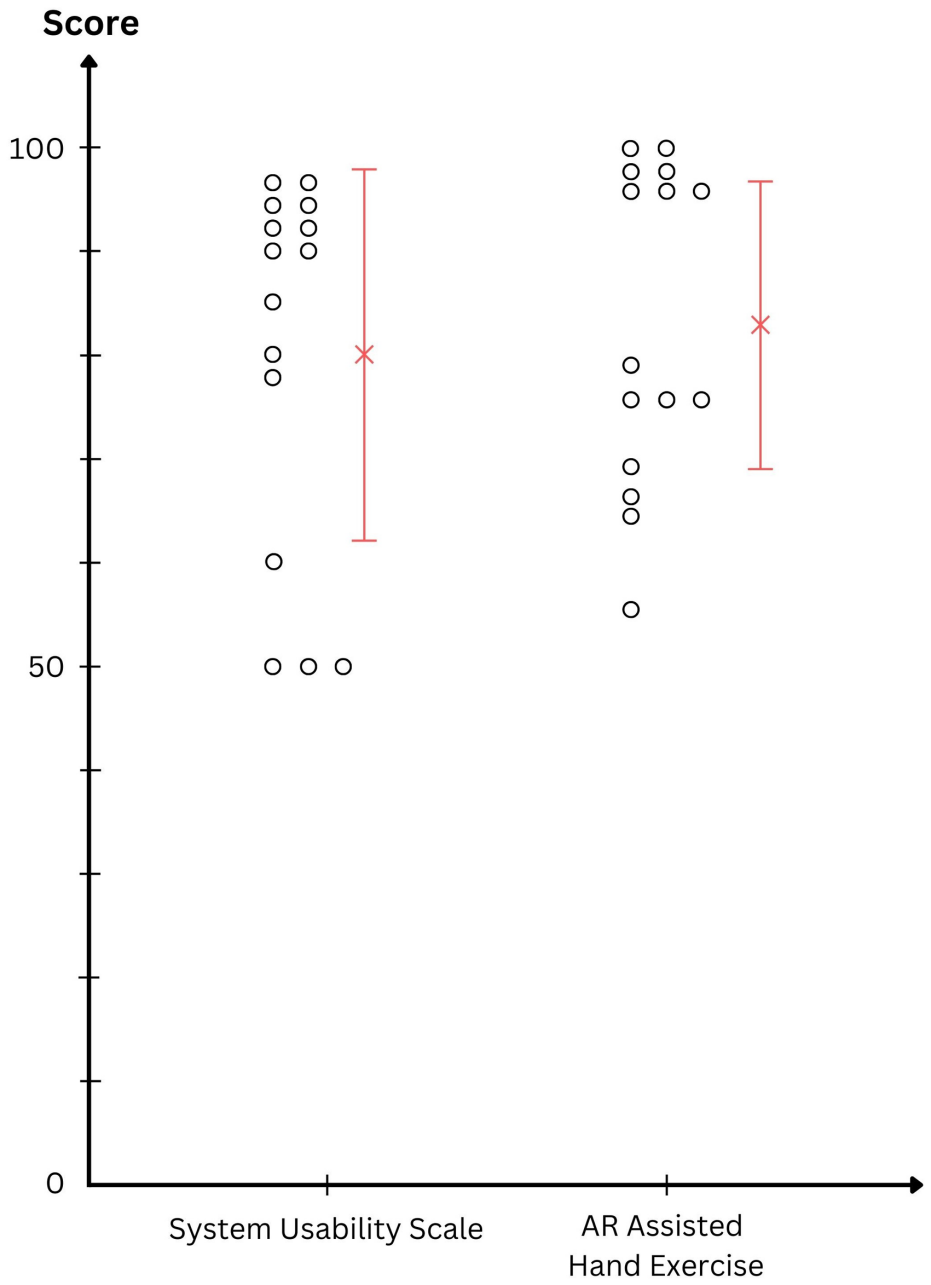
System Usability Scale scores and the performance scores of the CTS patients in the MR-assisted hand exercise program.

Due to logistical reasons, another participant group with similar diagnosis performed the MR-assisted hand exercise. Their performance scores are shown in Figure 6. They all performed above average (i.e., 50) as calculated in Materials and Methods based on the time they could hold each hand pose. The mean and standard deviation of the performance score was 83.3 ± 14.8, which is significantly greater than an average performance [one-tailed *t*(14) = 8.71; *p* < 0.001].

After the hand exercise was over, the participants completed the SUS questionnaire with an average result as 80.2 ± 18.3 (Figure 6). A SUS score above 68 is considered as better than an average system in the literature. The SUS results are statistically higher than this criterion [one-tailed *t*(14) = 2.57; *p* = 0.010]. According to the published distribution (Brooke, 1996), average SUS score in this study lies between the 85–89th percentile. The association between exercise performance and SUS score may show how much the overall results are dependent on the participants. Pearson correlation shows a significant association between the two columns of data in Figure 6 (ρ = 0.89, *p* < 0.001). It implies that, within this group of participants, those who found the MR-assisted hand exercise program better as a usable system also performed higher in the exercises.

## Discussion

### Methods for diagnosis of CTS

Medical ultrasound imaging and nerve conduction studies are both proven techniques for the diagnosis of CTS. Elnady et al. (2019) used high resolution ultrasound (HRUS) to assess median nerve cross-sectional area, and they reported that HRUS is a promising tool that can identify CTS and its severity with good accuracy. According to Georgiev et al. (2018), the sonographic methods have a number of benefits, such as being readily available, noninvasiveness and relatively lower cost. They simplify the diagnosis of CTS and shorten the evaluation time, and further provide several parameters regarding microanatomy, blood flow, and mobility. The median nerve typically gets bigger at the distal wrist crease in people with CTS symptoms (Wiesler et al., 2006). Yoshii et al. (2020) have argued that, with current advances, ultrasound measurement of the median nerve cross-section area may eliminate the need for electrophysiological testing in the future.

Quantitative Sensory Testing (QST) methods have also been applied in the clinical setting (Chong and Cros, 2004). These can target both small- and large-fiber pathways by using light touch, vibration, thermal, and pain stimuli. Due to availability, ease of application, and low cost, Semmes-Weinstein monofilaments (or von Frey hairs) were frequently used for testing light touch (Koris et al., 1990). Although the monofilaments can be calibrated and applied either manually or automatically with the help of a driving mechanism, skin-probe coupling cannot be as stable as electrodynamic mechanical shakers which can usually produce any mechanical waveform on the skin surface (Güçlü and Bolanowski, 2005; Güçlü and Öztek, 2007). However, such laboratory-grade equipment is typically bulky to achieve the desired scientific accuracy and precision. Given some technical (and psychophysical) trade-offs, smaller voice coils adopted in the Cortical Metrics device (Holden et al., 2012) allow reproducible spatial and temporal protocols for tactile QST (Güçlü et al., 2015). The biggest advantage of the mid-air haptics device, in comparison with the previous technology used for QST, is non-contact testing, thus somewhat eliminating the skin-probe coupling problem as long as the transducer distance can be maintained with a stable mechanical setup and tracking by cameras. Furthermore, the device can be used in any orientation in space and is applicable to all body sites (eye protection can be worn for face). Establishing a reproducible skin-probe coupling would require more effort if mechanical shakers are used at variable orientations, which would complicate QST in the clinic. Nevertheless, the stimulus control by the mid-air haptics device was not found yet to be adequate for QST (see limitations below) and needs further development.

Although the mid-air haptics technology is not yet ready to compete with established methods for the diagnosis of CTS, especially ultrasound imaging, it may be useful for online monitoring and home applications through cloud services under the supervision of physicians. The portability of the device allows for remote measurements, which would be especially efficient for follow up. The output of the device, with the API version used here, was not high enough to measure the affected hand’s index finger threshold, but normal hand’s thresholds and the affected hand’s thenar threshold could be reliably measured. This shows that even with mild CTS, the affected hand’s index finger threshold is elevated. If the above technical limitation can be remedied, the mid-air haptics device may be an alternative to tedious electrophysiological testing, especially of Grade 1 CTS patients who require more sensitive tests. Early verification of CTS, either at home or at the clinic, is beneficial to start an early treatment.

### VR-, AR-, and MR-assisted exercise programs

Tactile stimulation by mid-air haptics technology has been previously implemented as tactile feedback in medical training simulators (Balint and Althoefer, 2018). It is generally considered that such tactile feedback for improving palpation experience can increase user experience with low cost. Similar technology can be used to generate Braille code which visually impaired individuals can read with 90% accuracy (Paneva et al., 2020). Here, we used tactile feedback mainly for guiding the participants for hand position and also for motivation through the hand exercise program. The major contribution, however, was the hand tracking part of the mid-air haptics device.

There are many studies in the literature involving VR-assisted exercise programs. They usually enhance user experience by implementing VR on top of a traditional exercise scheme. For example, Cho and Sohng (2014) showed that a VR exercise program improves physical fitness, body composition, and fatigue more in hemodialysis patients, compared with the more traditional methods. Exercise with games also provides significant behavioral and physiological benefits to children with autism, despite the fact that many of these children lead sedentary lifestyles (Finkelstein et al., 2013). According to their results, most of the participants showed intense levels of effort and they stated that they would exercise more if they had access to these types of exercise games more. Elderly people can benefit from VR exercises to improve balance and gait (Park et al., 2015); sway lengths decreased compared to traditional ball exercises. On the other hand, VR-assisted exercise biking sessions yield higher levels of perceived exertion rates, confidence, and enjoyment compared with traditional sessions among college students (Zeng et al., 2017). Given the huge corpus of studies showing benefits, at least regarding motivation and engagement, the use of VR, AR, and MR in exercise programs has pretty much gained well-deserved popularity.

Here, we used the hand tracking software of the mid-air haptics device to evaluate sequential hand poses in an exercise program based on AAOS (2022). There has been limited research for applying similar technology to CTS. According to our knowledge, combining hand tracking with tactile/haptic feedback is a novel technique for preliminary diagnosis and rehabilitation of CTS. Although we could not assign the exercise program repetitively and study the treatment outcome, the participants found the MR-assisted program useful and scored high (>80% on average). This shows that they could follow the instructions and complete the hand poses very successfully. Furthermore, the average SUS score was also quite high (within the 85–89th percentile), which suggests that the system is user-friendly, time-saving, and capable of effectively satisfying the requirements defined by its users. As such, the system can provide higher engagement and sustained use, which is a key point for the CTS therapy. Sustaining the daily hand exercises can prolong the time to surgery and may even be capable of improving the symptoms in some patients. Just like the sensitivity measurement, the exercise program can be a part of cloud services, in which the physician can track the progress of each patient and take appropriate action. Such novel services are promising to give a significant impact on society, as well as the diagnosis and treatment of CTS patients.

### Limitations and future work

The main technical limitations of this study are regarding the use of ultrasound for vibrotactile stimulation and specifications of the mid-air haptics devices. First, stimulus control is not as good as mechanical shakers. The minimum diameter of the focus point is ~9 mm. Stimulation of such larger areas would recruit many mechanoreceptors, and thus may enhance sensitivity especially at high modulation frequencies (>100 Hz) due to spatial summation by the Pacinian psychophysical channel (Güçlü, 2021). Furthermore, the focus point has an ellipsoid shape at the vertical central line (Rakkolainen et al., 2020), and the shape elongates in other directions due to stray energy away from the focus. Therefore, small differences in hand location may change sensitivity measurements. In order to prevent this as much as possible, a custom-made stand was used with instructions to keep the hand still. In future work, the 3D shape of excitation may be optimized by tracking the hand quicker and concentrating the energy at the skin surface. In the current psychophysical experiment, we did not implement hand surface tracking because the API was not suitable for changes at that spatial and temporal scale.

Although there are ongoing studies to measure and to model the mechanical displacements on the skin surface due to ultrasonic excitation, the results as yet are not conclusive. We assumed that the vibrotactile stimulus frequency is equivalent to the frequency of the modulating wave. However, harmonic frequencies exist in the actual displacements (Chilles et al., 2019) which may also be dependent on the output level. Since the interface between the air and the skin has a large acoustic impedance mismatch, much energy is expected to be wasted. As a matter of fact, 324 transducers only produce a force of around 0.016 N (Hoshi et al., 2010). Combining many transducers increases the force, but with diminishing return. Therefore, developments in new transducer technology may significantly improve mid-air haptics devices. Ito et al. (2018) used transducers with different carrier frequencies (40 and 70 kHz) to increase the total force output. Overall, reliable calibration methods will be helpful to increase the application areas of ultrasonic tactile sensations.

Another limitation of the mid-air haptics technology is the working space. Optimum effects are obtained ~20 cm away from the transducer surface in the current device. Distance has largely an asymmetric relationship; distances below 20 cm are not operable, those above cause decreases in strength and accuracy of the haptic effect. This may also be improved with novel transducers. The output limitation mentioned in the current study was considered to be mainly due to the API of the particular device, over which we had no control of. According to the much recent announcements by the manufacturer, this issue seems to be partially remedied and the transducers are improved for future implementations.

On the other hand, the hand tracking part of the device was found to be quite adequate for the MR-assisted hand exercise program presented herein. The custom software and scoring method developed for this application resulted in a correlation between the exercise performance and SUS scores. This shows that the quality of the exercise creates a bias for the exercise system. Good performers like the usability, but bad performers do not. This is indeed not desirable from a system point of view and it affords future improvements in the hand exercise program. Nevertheless, the tactile/haptic feedback was found useful by the participants; we plan to enhance its effect to increase the motivation and the effort of the patient.

## General conclusion

As described in the electrodiagnostic criteria, sensory fibers are affected first in CTS. Therefore, the participant group in this study was particularly suitable for investigating tactile sensitivity as a means for early diagnostics. The main hypothesis about the thenar eminence is supported by the current results; 250-Hz vibrotactile thresholds of affected and healthy hands are very similar. This finding is most probably due to the branching of the median nerve (PCBm) before entering the carpal tunnel (Smith and Ebraheim, 2019). On the other hand, according to some studies in the literature, PCBm is also affected in CTS due to accompanying causes. Therefore, an electrodiagnostical exam of the PCBm is not traditionally considered for comparison, especially in later stages of CTS. According to Uluc et al. (2015), 56% of CTS patients may have abnormal nerve conduction also in PCBm. This finding was somewhat replicated by Rathakrishnan et al. (2007) who reported that percentage as 46%. Eventually both nerve branches seem to be injured in CTS. However, since our experimental group had mild CTS, PCBm might have been spared in this study.

Although we could not reach a numerical result regarding 250-Hz vibrotactile thresholds at the index fingers of the affected hands because of device limitations, the psychophysical tracking procedure implies that they are well above 1 au. This is because, if they were below that limit, the procedure would most likely converge. Since the average threshold at the index finger of the healthy hand is 0.90 au, one may conclude that vibrotactile thresholds at the affected index fingers are elevated in mild CTS. In conclusion, mid-air haptics technology appears to be a viable tool for early diagnosis of CTS, if certain improvements are made in hardware and software, and especially regarding calibration.

Mid-air haptics combined with hand tracking offers a novel platform for MR-assisted hand exercise programs. These would be especially useful for the rehabilitation of CTS patients. The current study shows that the device used for that purpose has adequate specifications regarding hardware and software, and can motivate the patients for sustained exercise. Applying this technology to cloud services for remote care seems to be currently feasible as well.

## Data availability statement

The raw data supporting the conclusions of this article will be made available by the authors, without undue reservation.

## Ethics statement

The studies involving humans were approved by Clinical Research Ethics Committee of the Health Sciences University Kanuni Sultan Süleyman Training and Research Hospital (application number: 2021.06.196). The studies were conducted in accordance with the local legislation and institutional requirements. The participants provided their written informed consent to participate in this study.

## Author contributions

MA: Conceptualization, Data curation, Formal analysis, Investigation, Methodology, Resources, Software, Validation, Visualization, Writing – original draft, Writing – review & editing. AM: Conceptualization, Funding acquisition, Methodology, Project administration, Resources, Supervision, Validation, Writing – review & editing. HS: Data curation, Investigation, Methodology, Resources, Writing – review & editing. BeG: Software, Visualization, Writing – review & editing. MK: Project administration, Resources, Writing – review & editing. BuG: Conceptualization, Funding acquisition, Methodology, Project administration, Resources, Supervision, Validation, Writing – review & editing.
